# Evaluation of Biochemical Composition of Amniotic and Allantoic Fluids at Different Stages of Pregnancy in Queens

**DOI:** 10.3390/ani12111414

**Published:** 2022-05-30

**Authors:** Enrico Bigliardi, Matteo Rizzi, Mara Bertocchi, Laura Denti, Carla Bresciani, Alessandro Vetere, Francesco Di Ianni

**Affiliations:** Department of Veterinary Medicine, University of Parma, 43123 Parma, Italy; enrico.bigliardi@unipr.it (E.B.); mara.bertocchi@unipr.it (M.B.); laura.denti@unipr.it (L.D.); carla.bresciani@unipr.it (C.B.); alessandro.vetere@unipr.it (A.V.); francesco.diianni@unipr.it (F.D.I.)

**Keywords:** cat, fetal fluids, pregnancy, amnion, allanto-chorion

## Abstract

**Simple Summary:**

Fetal fluid contents influence growth and development, fetal wellbeing and a protection mechanism for the fetus itself. Very little is known about the composition of fetal fluids during pregnancy in cats and about changes in contents that occur throughout gestation. In this study, initial results regarding the biochemical composition of fetal fluids are provided. A comparison with maternal serum was also performed. A complete understanding of trends can be helpful for the early detection of some pathologies during pregnancy, such as in humans and mares.

**Abstract:**

Fetal fluid contents have functions in protecting fetuses and are essential for fetal development and maturation. However, little is known about the exact physiological functions of fetal fluids in fetal development, as well as the changing composition throughout the gestational period in cats. In this study, the biochemical composition of amniotic (AMN) and allantoic (ALL) fluids was investigated, as well as in the maternal serum of pregnant queens. Eighteen queens were included in this study and assigned to six different groups, D_20_, D_25_, D_30_, D_40_, D_45_ and D_60_, according to the gestational stage of fetal development. A total of 44 amniotic and 37 allantoic samples were collected. Fetal fluids contained lesser concentrations of alanine aminotransferase, albumin, cholesterol, triglycerides, creatine kinase, amylase, total protein and globulin than maternal serum. Other variables, such as aspartate aminotransferase, gamma-glutamyl transferase, bilirubin and alkaline phosphatase, were in different concentrations at specific stages of gestation when compared to maternal serum. There were no differences between fetal fluids and maternal serum for lactate dehydrogenase, urea, lipase or glucose concentrations. There were greater concentrations of creatinine in amniotic fluid than in allantoic fluid or maternal serum. Based on the results of this study, fetal fluids do not accumulate as a result of the simple filtration of maternal blood, but rather, the fetus produces many of these components as a consequence of organ development and maturation.

## 1. Introduction

Fetal fluids are contained within membranes that are essential for metabolic, gaseous and hormonal exchange [1]. These fluids also function to protect embryos from physical damage and are essential for the safety of the conceptus. Each membrane, the amniotic and the allantoic, originates from different structures: the amniotic membrane arises from folds that, during the progression of pregnancy, merge with each other until the amniotic sac is complete. At this point, the amniotic membrane is composed internally of the ectoderm and externally of the somatopleura [2]. In contrast, the allantoic sac originates from the caudal region of the gut and develops, occupying the extraembryonic coelom until it distends into the remaining unoccupied space [2].

Additionally, fetal fluids originate from different sources: amniotic fluid (AMN) accumulates from secretions from the respiratory tract, oral cavity, gastrointestinal tract and fetal skin before keratinization [3], providing essential nutrients and other factors for the developing fetus [4]. In humans, the biochemical composition of AMN is very similar to the extracellular fluid of the fetus at the beginning of pregnancy, probably due to transudation across the unkeratinized skin [5]. A similar phenomenon has been described in sheep, which has an important exchange of water and electrolytes across the skin [6].

Allantoic fluid (ALL), however, accumulates from mesonephros, metanephros and kidney secretions, while as the gestational period progresses, fetal urine is diverted into the amniotic fluid through the urethra due to the occlusion of the urachus [7].

In humans, the composition of both AMN and ALL is well documented, while in animals evaluated by veterinary practitioners, far less is known. In cattle [8] and sheep [9], concentrations of fetal fluids have been quantified and analyzed. A recent study with pregnant dogs [10] reported the first results of the biochemical and electrolyte composition of fetal fluids at the time of the parturition.

The objective of the present study was to determine the biochemical composition of both amniotic and allantoic fluids from feline embryonic vesicles from day 20 to 60 of the gestational period. Additionally, there was a comparison of concentrations and contents to that of AMN and ALL maternal serum (MS).

## 2. Materials and Methods

### 2.1. Animals

For this study, a total of 18 pregnant queens were submitted to the obstetrics unit at the Teaching University Veterinary Hospital (OVUD) of the Department of Veterinary Medicine of the University of Parma. The total number of amniotic samples was 44, while the total number of allantoid chambers was 37. All queens were stray cats that had been delivered to the hospital for the population control program of the Emilia-Romagna Region. All cats were clinically healthy on physical examination, and the stages of gestation were estimated to be between 20 and 60 days.

### 2.2. Surgical Procedure

All animals were premedicated with a combination of ketamine (5 mg/kg; Lobotor^®^, Acme S.r.l., 42025 Corte Tegge-Cavriago (RE), Italy), dexmedetomidine (7 mcg/kg; Dexdomitor^®^, Vetoquinol Italia S.r.l., 47032 Bertinoro (FC), Italy) and butorphanol (0.2 mg/kg; Nargesic^®^, Acme S.r.l., 42025 Corte Tegge-Cavriago (RE), Italy) administered intramuscularly and were then intravenously administered with propofol (3 mg/kg; Proposure^®^, Boehringer Ingelheim Animal Health Italia S.p.A., 20139 Milano, Italy). A prophylactic injection of a combination of benzylpenicillin + streptomycin (2 mL/10 kg b.w.; Neotardocillina^®^, Vetoquinol Italia S.r.l., 47032 Bertinoro (FC), Italy) was administered intramuscularly, and a single dose of robenacoxib (2 mg/kg; Onsior^®^, Elanco Italia S.p.A., 50019 Sesto Fiorentino (FI), Italy) was administered subcutaneously. Lidocaine spray was applied to the arytenoid cartilage, and then the cats were intubated; anesthesia was maintained with isoflurane (1.5–2%) in oxygen. Ovariohysterectomy was conventionally performed through a ventral midline laparotomy. As soon as the ovaries and the gravid uterus were removed, the embryonic vesicles were measured and processed within 10–15 min to collect AMN and ALL samples.

### 2.3. Sample Collection

**Figure 1:**
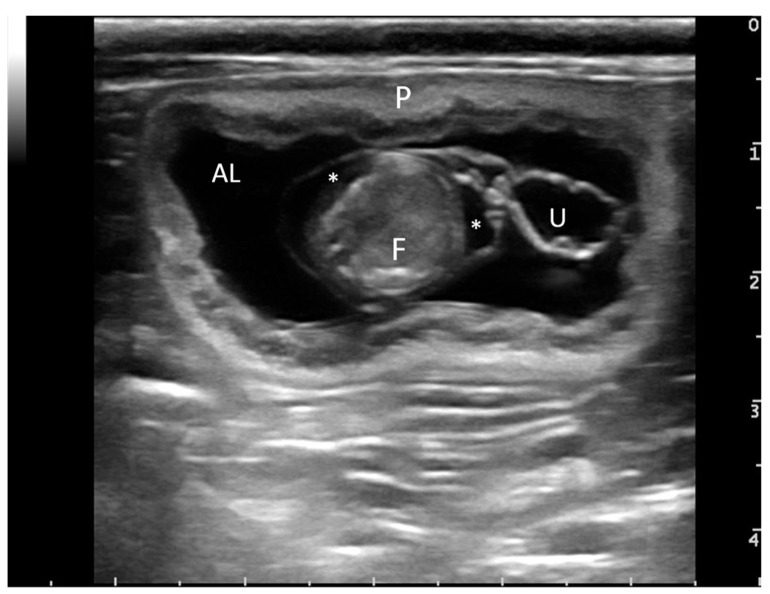
**Ultrasonographic examination of an embryo vesicle.** Placenta is marked with “P”, allantoic sac with “AL”, amniotic sac with “*”, fetus with “F” and umbilical cord with “U”.

Queens were assigned to six groups according to gestational age, determined by measuring the diameter of the embryonic vesicles [11] and the ultrasonic examination of the vesicles [12]. The ultrasonic examination was performed using a MyLabTM30Gold scanner (Esaote, Florence, Italy). According to [11,12], vesicles of approximately 19–22 mm in diameter corresponded to a gestational age of about 20 days (D_20_), vesicles of 27–30 mm to a gestational age of around 25 days (D_25_) and vesicles of 34–37 mm to approximately 30 days of gestation (D_30_). After the first month of pregnancy, vesicle diameters are difficult to measure ultrasonographically, so a different method of classification was adopted. *Zambelli* et al. [12] also described the gestational age in reference to the abdominal diameter of the fetus: a value of approximately 1.8 cm corresponded to about day 45 of gestation (D_45_), and a diameter of approximately 3.8 cm corresponded to the time when parturition occurred (D_60_). For day 40 of the gestational period (D_40_), the fetal age was determined based on the appearance of the gut using ultrasonic procedures and the division of the vertebrae [12], as well as the experience of the clinicians. Ultrasonographically, during the mid-gestational period, both allantoic and amniotic sacs, as well as the fetus and the umbilical cord, were easily detected (Figure 1). Precision in determining the gestational period without knowing the exact day of ovulation or mating is complex. In a breeding condition, knowing the exact day of mating and the ovulation induced by mating, it is possible to establish the day of pregnancy with more precision. In this study, queens were stray cats, so the day of mating was unknown. Therefore, the moment of gestation has been included in a range of several days, relying on diameters and biometric surveys, according also to *Zambelli* et al. [12]. In fact, the number of gestational chambers, the size and the breed of the queen can influence the growth rate and the dimension of the vesicles. According to vesicles size detected, a period of gestation has been defined: D_20_ corresponded to days 15–21 of pregnancy, D_25_ to days 22–28, D_30_ to days 29–35, D_40_ to days 36–42, D_45_ to days 43–49 and D_60_ 57–63.

Group D_20_ included 7 AMN samples and 0 ALL; group D_25_ included 7 AMN and 7 ALL samples; group D_30_ was composed of 7 AMN and 7 ALL samples; group D_40_ included 7 AMN and 7 ALL; group D_45_ included 8 AMN and 8 ALL; and D_60_ included 8 AMN and 8 ALL samples.

Before surgery, a blood sample was collected from each queen from the jugular vein into plastic tubes containing Li-Heparin and samples were analyzed immediately. The data obtained were evaluated using the operative system of the structure, and all blood values of the maternal serum were within ranges, albeit there were homeostatic variations as anticipated.

**Figure 2:**
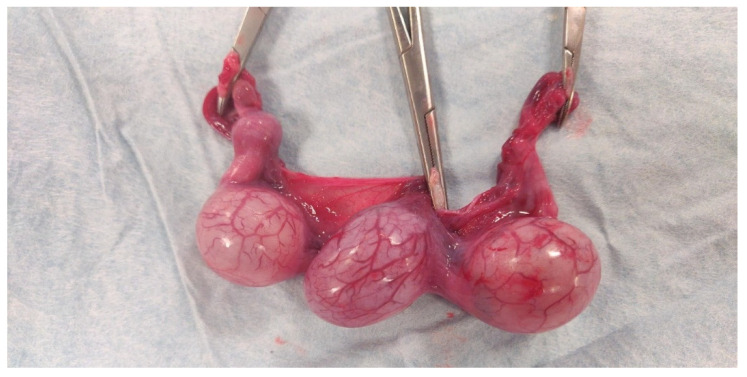
**Uterus after ovariohysterectomy.** Three different embryonic vesicles can be seen.

**Figure 3:**
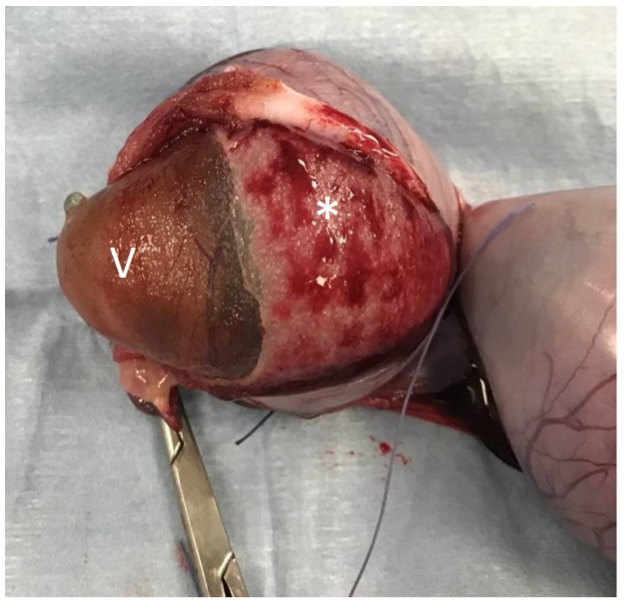
**Dissection of an embryonic vesicle from the uterus.** Embryonic vesicle is marked with “V” and the chorionic membrane with “*”.

**Figure 4:**
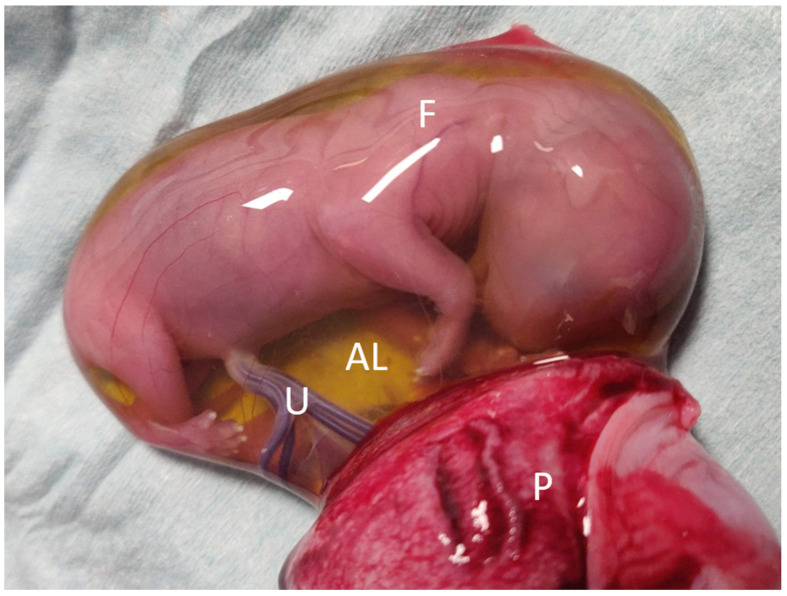
**Excision of an embryonic vesicle.** Visualization of the allantoic sac. Placenta (marked with “P”) has been carefully dissected from the rest of the vesicle. Allantoic sac (“AL”) is filled with a yellow fluid and contains the fetus (“F”). The umbilical cord is recognizable and is marked with “U”.

**Figure 5:**
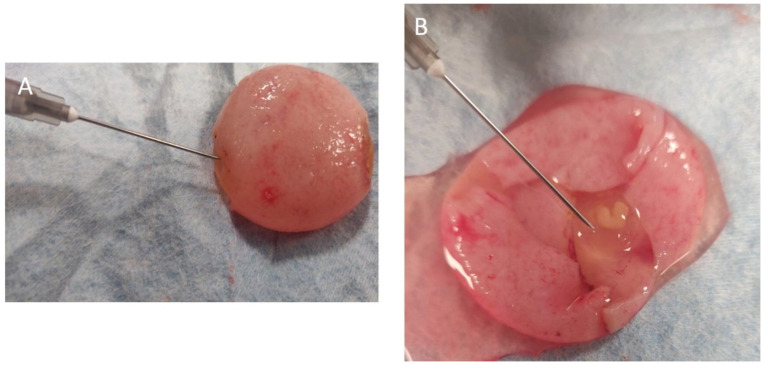
**Withdrawal of fetal fluids.** The fluid collection was made using a 23G needle connected with a 2.5 mL syringe. (**A**) Sampling of allantoic fluid from a pregnancy of about 20 days. (**B**) Collection of the amniotic fluid from the same embryonic vesicle.

After ovariohysterectomy was performed (Figure 2), the embryonic vesicles were carefully dissected from the uterus (Figure 3 and Figure 4). Allantoic fluid was collected initially using a 23G needle connected to a 2.5 mL syringe (Figure 5A). Subsequently, the embryos and amniotic sacs were exposed, and AMN was aspirated using a 23G needle connected to a 2.5 mL syringe (Figure 5B). Fluid samples were frozen immediately after collection and stored at −80 °C until analyses were performed. This study was approved by the ethical commission of the University of Parma.

### 2.4. Biochemical Analysis

Biochemical parameters analyzed in MS, AMN and ALL samples included albumin (ALB), alkaline phosphatase (ALP), amylase (AMY), bilirubin (BIL), cholesterol (CHOL), creatinine (CREA), creatine kinase (CK), gamma-glutamyl transferase (GGT), globulin (GLO), glucose (GLU), aspartate aminotransferase (AST), alanine aminotransferase (ALT), lactate dehydrogenase (LDH), lipase (LIPA), total protein (PT), triglycerides (TRIG), urea (UREA) and specific gravity (SG). Biochemical analyses were performed using a Cobas Integra^®^ 400 Plus (Roche Diagnostics International AG; Risch-Rotkreuz, Switzerland) and Cobas C pack reagents (Roche Diagnostics S.p.A.; Monza (MB), Italy) for all variables considered. The data are reported in Table 1. In the table, there are also reported data depicted by *Veronesi* et al. [10] on at-term physiological pregnancies in dogs.

### 2.5. Statistical Analysis

Results were analyzed using the SAS statistical package. The LS-MEANS procedure was used to list mean differences. Data obtained were first analyzed by comparing the three groups of samples, AMN, ALL and MS, at different stages of pregnancy. Moreover, data from every group of samples were analyzed among the different stages of pregnancy for every group to detect variations during the gestational period. Significance was assumed with *p* < 0.05.

## 3. Results

### 3.1. Liver Enzymes

**Figure 6:**
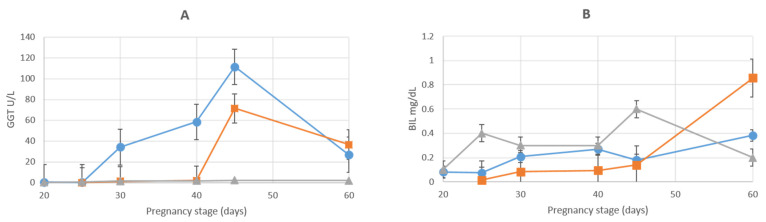
Graphic representation of GGT (**A**) and BIL (**B**) concentrations during pregnancy in maternal serum (marked with ▲), amniotic fluid (marked with ●) and allantoic fluid (marked with ■). Values are indicated as mean ± SE.

There were no marked changes in alanine aminotransferase throughout the gestational period, and values were less in the AMN and ALL compared to those in the MS. Additionally, the concentration of ALB was less than in the MS, although, in both the AMN and ALL, values tended to increase as the gestational period progressed. At D_60__,_ concentrations of ALB were greater in the AMN than in ALL (*p* < 0.05). Concentrations of AST in the AMN and ALL, however, were less than in the MS, but there was a notable increase subsequent to D_45_, especially in the ALL, with values similar to those in the MS during the first half of the gestational period. Concentrations of AST on D_40_ were different (*p* < 0.05). Concentrations of GGT in the AMN and ALL were comparable to those in the MS until D_25__,_ when concentrations of GGT started to increase in the AMN. Concentrations of GGT in the ALL were similar to those of the MS until D_40_. Concentrations in the ALL also started to increase. At D_45_, concentrations of GGT in both the AMN and ALL were maximal and then began to decrease until D_60_ (Figure 6A). At D_30_ and D_40__,_ there was a difference in the concentrations of GGT between the two fetal fluids (*p* < 0.05). Concentrations of BIL in the MS increased in a two-phase pattern, first from D_20_ to D_25_ and then from D_40_ to D_45_, and subsequently, there was a gradual decrease in BIL until D_60_. In the ALL, there was a marked increase in BIL after D_45_ of the gestational period, and in the AMN, there was also a slight increase in BIL concentrations, with maximal values in both the AMN and ALL at D_60_ (Figure 6B). Concentrations of LDH in the MS were similar throughout the gestational period; however, there were changes in the fetal fluids. In the AMN at D_20_, there was the maximal value for LDH, and concentrations subsequently decreased until D_45,_ when concentrations began to increase; however, there were relatively greater concentrations of LDH in the ALL on D_30_ and D_45__,_ when concentrations started to increase and were maximal at D_60_.

### 3.2. Kidney Enzymes

**Figure 7:**
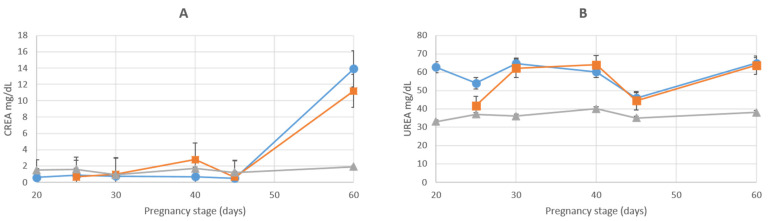
Graphic representation of CREA (**A**) and UREA (**B**) concentrations during pregnancy in maternal serum (marked with ▲), amniotic fluid (marked with ●) and allantoic fluid (marked with ■). Values are indicated as mean ± SE.

Concentrations of CREA in the AMN continued to be similar until D_45_, when they started to increase until D_60_. Allantoic concentrations of CREA increased slightly around D_40,_ and there was a marked increase from D_45_ to D_60_. Except for the latter gestational period, the concentrations of CREA in the ALL and AMN were similar to those in the MS (Figure 7A). Concentrations of UREA in the MS were similar throughout pregnancy and remained at these lesser concentrations for the rest of the gestational period in both fetal fluids. Amniotic UREA concentrations decreased until D_25_ and then increased until D_30_ while allantoic UREA concentrations increased; subsequently, there was a similar pattern for the changes in concentrations until the end of gestation with increases until D_40_ and subsequent decreases until D_45_, with one last increase until D_60_ (Figure 7B). In the statistical analyses, amniotic UREA concentrations were greater (*p* < 0.05) than allantoic UREA concentrations on D_25_ and D_40_.

### 3.3. Pancreatic Enzymes

There was an increasing trend in both AMY and LIPA throughout the gestational period. In particular, concentrations of AMY were always less than those in the MS throughout all the stages of development. Subsequent to D_45_, concentrations of AMY and LIPA in the ALL increased markedly but continued to be lower than concentrations in the MS. There was a difference (*p* < 0.05) in the concentrations of AMY and LIPA between the AMN and ALL during most of the gestational periods, except at D_45_. The pattern of concentrations of LIPA during the gestational period, however, was different from concentrations in the AMN, increasing until D_45_ when concentrations were maximal, and there was a subsequent marked decrease until D_60_. Otherwise, LIPA concentrations in the ALL were slightly less than those in the AMN for all animals. The concentrations of LIPA in the AMN, however, were greater between D_45_ and D_60_, with the concentrations being comparable to those in the MS. Of note, on D_30_, the concentrations of LIPA in the ALL were less (*p* < 0.05) than those in the AMN.

### 3.4. Muscular Enzymes

**Figure 8:**
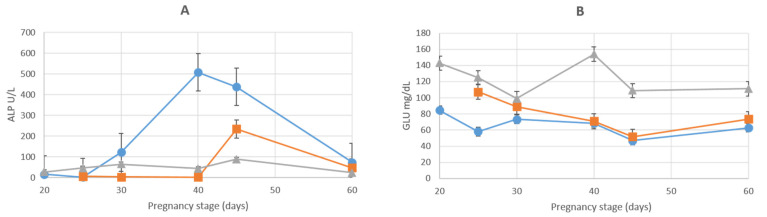
Graphic representation of ALP (**A**) and GLU (**B**) concentrations during pregnancy in maternal serum (marked with ▲), amniotic fluid (marked with ●) and allantoic fluid (marked with ■). Values are indicated as mean ± SE.

The concentration of CK was consistently less in both the AMN and ALL when compared to those in the MS and was similar throughout the gestational periods. There was an increase in ALP concentrations in the AMN until D_40,_ when concentrations started to decrease, especially after D_45_. There was an increase in ALP concentrations in the ALL from D_40_ to D_45_, after which there was a decrease until D_60_ (Figure 8A). Concentrations of ALP in the AMN were greater (*p* < 0.05) than in the ALL at both D_30_ and D_40_.

### 3.5. Lipid Metabolism Enzymes

The concentrations of TRIG and CHOL were less than MS in both the AMN and ALL. The concentrations of CHOL were similar throughout the gestational period, while the concentrations of TRIG increased in the AMN subsequent to D_45_.

### 3.6. Total Proteins, Glucose and Globulins

The total protein concentrations were always lower in the AMN and ALL than in the MS; however, the concentrations in both the AMN and ALL increased subsequent to the D_45_ of the gestational period. The concentrations of globulin were similar at all stages of the gestational period, except for D_60_ when an increase occurred, and were less than those in the MS. The pattern of change in the concentrations of GLU, however, was similar to that in the MS, decreasing until D_45_, and subsequently, there was a slight increase until the end of the gestational period (Figure 8B). The concentrations of GLU in the AMN were less (*p* < 0.05) than in the ALL from D_25_ to D_40_.

### 3.7. Specific Gravity

Specific gravity values were similar until D_45__,_ when values for both the AMN and ALL subsequently increased throughout the rest of the gestational period. Values for SG in the ALL were greater than those in the AMN. On D_25_, values for the SG of the AMN were less (*p* < 0.05) than those of the ALL.

## 4. Discussion

The results of this study revealed that the composition of feline fetal fluids is not a result of simple filtration from maternal blood, as indicated by *Fresno* et al. [13]. The fetus has important functions in modulating the final fluid composition throughout the gestational period. Results from the present study of feline AMN and ALL compositions indicate there were similar compositions in the two fetal fluids. Throughout the gestational period, the AMN is poorly vascularized, but these vessels are in close proximity to the ALL membrane and AMN cells, making diffusion possible [14]. Furthermore, modifications during pregnancy are likely a reflection of changes in metabolic processes and transport activity and differential contributions of the placental and fetal tissues to AMN and ALL compartments [7]. Results from a previous study performed on sheep indicated that changes in concentrations of constituents in the ALL composition changed throughout the gestational period, while the concentrations of these substances in the AMN remained relatively constant [9]. These findings were consistent with the changes in the concentrations of some but not all of the biochemical compounds analyzed in the present study.

From the results of the present study, UREA seems to accumulate in both the AMN and ALL in a similar pattern throughout the gestational period. This could indicate a possible equilibration mechanism between fetal compartments [13]. In contrast, CREA accumulates in the ALL only at the end of the gestational period. Most likely, CREA secreted from the fetus does not easily cross into the maternal blood and, therefore, accumulates in fetal fluids [7], while it is also possible that it is transported into the ALL fluid because fetal urine in cats accumulates in the allanto-chorion cavity [2]. Urea and creatinine concentrations were distinct from those of dogs [10], whereas concentrations of both were higher in the ALL than in AMN.

Many tissues and cell types have ALP functions, such as cells of the liver, kidneys, intestinal mucosa and placenta [15]. In the feline embryo, there was the greatest ALP activity subsequent to the D_25_ of the gestational period in the AMN and subsequent to the D_40_ in the ALL. This is likely because fetal ossification occurs during this period. In all young animals, there is greater ALP activity than in adults, with values as much as 10-fold greater than those of blood values [15].

Concentrations of AMY in feline fetuses are less than in the MS. This is in contrast to humans, where fetal AMY has only slightly lesser concentrations than in the MS [16], and in dogs, where there is no difference in AMY concentrations between the two fetal fluids [10]. These findings indicate that the pancreas has lower AMY activity because very little digestion occurs during fetal life. Both AMY and LIPA, however, increase in the ALL toward the end of the gestational period, likely due to the fetal renal maturation, which leads to the filtering of small-molecular-weight enzymes.

The GGT enzyme has marked functions in transporting amino acids across cell membranes. In domesticated animals and humans, GGT is present in small concentrations in circulating blood because the primary functions of this enzyme are in the cells inside the liver, pancreas and other organs. During fetal life, GGT concentrations may increase in both the AMN and ALL due to fetal growth and the production of proteins and use of amino acids or due to the accumulation of BIL inside fetal membranes. In humans, the concentrations of GGT are greater than those in MS throughout pregnancy [16]. In contrast to values reported in dogs [10], GGT was in greater concentrations in the AMN than in ALL in feline samples.

The functions of AST are similar to those of GGT. This enzyme is involved in protein synthesis and the production of ammonia, but during fetal development, there is only an increase in AST during the last quarter of the gestational period. There may be functions of AST in fetal development, but it is plausible that AST is important in the production of ammonia for conversion into urea because the kidneys are fully formed after D_44_ of the gestational period [11]. These previous findings are consistent with the findings regarding UREA in the present study.

In the present study, BIL concentrations were greatest around the time of parturition, and concentrations were greater in fetal fluids than in the MS; these findings are consistent with those in studies with humans [16] and dogs [10]. The fetal liver synthesizes BIL and bile acids during the early stages of gestation when these are not required for digestive purposes [17]. It is possible that in cats, before D_40_ to D_45_ of the gestational period, BIL accumulates in the AMN, while after this period, the kidneys are fully functional, and BIL is in greater concentrations in the ALL. The findings in the present study are consistent with those of *Fresno* et al. [13] and are supported by the fact that, in cats, fetal urine accumulates in the allanto-chorion cavity [2].

Compared to the MS, in both fetal fluids, there were markedly fewer nutritional components, such as total proteins, glucose, TRYG and CHOL, which may suggest that these fluids are not an important source of fetal nutrition. In humans, the concentrations of these nutrients in the AMN are also less than in the MS [16], and in dogs, the concentrations of all four of these nutrients were greater in the MS than in AMN [10]. This may indicate that nutritional components are primarily derived from maternal blood and are secreted only in small quantities into fetal fluids.

Pregnancy is associated with insulin resistance in humans and dogs, which occurs in response to the suppression of the intracellular transport of glucose and a resulting increase in its concentration in blood [18]. In the present study, the glucose concentration in maternal serum did not undergo any marked alterations during the gestational period. This is indicative that queens are less susceptible than women and dogs to progesterone actions in modulating insulin concentrations.

In humans, an early analysis of fetal fluids is very useful to understand if there will be complications during the gestational period. The proteomic analysis of fetal fluids resulted in the diagnosis of inflammatory conditions such as placentitis [19]. A similar diagnosis can be observed in mares, where the up- or down-regulation of some components of fetal fluids can be indicative of a pathological condition [20]. Results from a recent study with buffalo indicated that some proteins can be in greater concentrations during the first stages of gestation, indicating that evaluations of the concentrations of these proteins can be used as a diagnostic approach for an early diagnosis of pregnancy [21].

## 5. Conclusions

Feline fetal fluids can no longer be considered to arise simply from maternal blood filtration. Instead, constituents of maternal blood are part of the composition of fetal fluids, but most of these constituents result from fetal physiological functions. The fetus is an important source for modulating the composition of both AMN and ALL fluids throughout the gestational period. Further studies are needed to better understand the correlation between the biochemical composition of fluids and fetal development and maturation and the changes that occur to fetal fluids throughout the gestational period.

There have been few reports of results from studies in which the composition of fetal fluids has been evaluated. Therefore, future studies focused on the evaluation of the possibility of an early diagnosis of pregnancy, as well as some pathologies during the gestational period, are warranted.

## Figures and Tables

**Table 1:** Concentration of biochemical components throughout pregnancy in amniotic fluid (AMN) and allantoic fluid (ALL). Values are expressed as mean ± SE. Number of samples: D_20_, AMN = 7, ALL = 0; D_25_, AMN = 7, ALL = 7; D_30_, AMN = 7, ALL = 7; D_40_, AMN = 7, ALL = 7; D_45_, AMN = 8, ALL = 8; D_60_, AMN = 8, ALL = 8. A comparison with physiological at-term pregnancies in dogs is performed (data depicted by Veronesi et al. [10]), data are represented in the “DOG” column.

	**D20**	**D25**	**D30**	**D40**	**D45**	**D60**	**DOG**
**ALBUMIN (G/L)**							
**AMN**	0.3 ± 0.1	0.09 ± 0.02	0.14 ± 0.05	0.16 ± 0.05	0.15 ± 0.05	0.49 ± 0.1	0.8 ± 0.05
**ALL**		0.1 ± 0.06	0.08 ± 0.07	0.1 ± 0.02	0.55 ± 0.15	0.86 ± 0.05	0.9 ± 0.05
**ALP (U/L)**							
**AMN**	15.8 ± 2.8	2.4 ± 0.5	121.7 ± 27.6	507.1 ± 9.6	437.2 ± 66.5	74 ± 7.9	126.0 ± 6.76
**ALL**		5.8 ± 2	3.9 ± 1.9	2.7 ± 0.6	234.1 ± 30.8	46.9 ± 6.5	25.7 ± 3.29
**AMY (U/L)**							
**AMN**	2.5 ± 0.1	2.88 ± 0.19	13.75 ± 6.3	13.3 ± 6.3	25.47 ± 5.5	64.3 ± 9.3	40.0 ± 3.01
**ALL**		7.2 ± 1.6	8.7 ± 7.3	8.4 ± 5.5	37.5 ± 9.7	172 ± 10.6	33.2 ± 1.9
**BIL (MG/DL)**							
**AMN**	0.08 ± 0.007	0.07 ± 0.005	0.21 ± 0.02	0.27 ± 0.04	0.18 ± 0.02	0.38 ± 0.6	0.2 ± 0.02
**ALL**		0.02 ± 0.005	0.08 ± 0.04	0.09 ± 0.05	0.14 ± 0.02	0.86 ± 0.04	0.6 ± 0.05
**CHOL (MG/DL)**							
**AMN**	0.67 ± 0.5	2.11 ± 1.01	2.33 ± 3.21	1.33 ± 1.53	1.47 ± 0.5	3.34 ± 0.58	3.3 ± 0.41
**ALL**		2.2 ± 0.8	0.7 ± 0.6	0.7 ± 0.5	5.2 ± 1	2.6 ± 0.5	3.7 ± 0.56
**CREA (MG/DL)**							
**AMN**	0.58 ± 0.01	0.84 ± 0.01	0.74 ± 0.23	0.68 ± 0.04	0.51 ± 0.04	13.93 ± 6.12	2.8 ± 0.15
**ALL**		0.65 ± 0.01	0.98 ± 0.4	2.8 ± 1.6	0.6 ± 0.07	11.2 ± 3.4	28.3 ± 2.33
**CK (U/L)**							
**AMN**	36.5 ± 2.2	5.3 ± 1.5	2.4 ± 1.1	2.7 ± 0.6	1 ± 0	5.7 ± 1.6	3.2 ± 0.41
**ALL**		13.5 ± 6	23.25 ± 25.6	7.7 ± 7.2	2.9 ± 1.6	35.4 ± 11.3	10.3 ± 1.72
**GGT (U/L)**							
**AMN**	0.5 ± 0.1	0.2 ± 0.04	34.2.5 ± 1.89	58.4 ± 10.72	111.3 ± 13.05	26.8 ± 4.67	3.7 ± 0.24
**ALL**		0.1 ± 0.05	1.1 ± 0.25	1.99 ± 1	71.4 ± 7	36.5 ± 2.2	59.8 ± 8
**GLO (G/L)**							
**AMN**	0.1 ± 0	0.1 ± 0	0.11 ± 0.01	0.1 ± 0	0.09 ± 0.02	1.08 ± 0.8	1.0 ± 0.19
**ALL**		0 ± 0	0.07 ± 0.05	0.4 ± 0.5	0.47 ± 0.1	2.7 ± 0.1	5.3 ± 0.46
**GLU (MG/DL)**							
**AMN**	84.5 ± 1.3	58 ± 2	73.3 ± 19.8	68.5 ± 1.38	47 ± 3	62.7 ± 3.5	24.5 ± 1.38
**ALL**		107.2 ± 4.5	89.1 ± 28.4	71 ± 13.7	51.6 ± 2.1	73.7 ± 4	26.6 ± 2.22
**AST (U/L)**							
**AMN**	7.3 ± 1.5	3.2 ± 0.7	1.7 ± 0.7	3 ± 3	4 ± 1	14.3 ± 1.6	9.4 ± 0.58
**ALL**		4 ± 1	3.4 ± 1.2	5 ± 2.6	3.4 ± 0.5	22.2 ± 0.7	11.4 ± 1.16
**ALT (U/L)**							
**AMN**	2.5 ± 1.3	1 ± 0	1.8 ± 0.7	1 ± 0	1 ± 0	1 ± 0	1.10 ± 0.11
**ALL**		0.9 ± 0.3	2.7 ± 2	4 ± 2.6	1.1 ± 0.1	1.4 ± 0.5	2.5 ± 0.8
**LDH (U/L)**							
**AMN**	382.5 ± 6.2	113.4 ± 10.2	21.5 ± 12.2	81.4 ± 50.7	12.2 ± 1.7	283 ± 18.7	29.8 ± 2.8
**ALL**		63.2 ± 5.5	82.6 ± 7.2	53.9 ± 5.5	26.9 ± 3.5	510 ± 23.1	116.6 ± 16.62
**LIPA (U/L)**							
**AMN**	2.6 ± 1.5	3.1 ± 1.6	8.5 ± 1.5	8.7 ± 1.5	22 ± 2	9.9 ± 1	5.8 ± 0.25
**ALL**		1.8 ± 1	3.2 ± 1	5 ± 1	17.7 ± 2.5	20.1 ± 1	11.6 ± 0.99
**PT (G/L)**							
**AMN**	0.4 ± 0.01	0.19 ± 0.02	0.25 ± 0.05	0.26 ± 0.05	0.2 ± 0.05	1.9 ± 0.3	1.5 ± 0.18
**ALL**		0.1 ± 0.05	0.2 ± 0.07	0.2 ± 0.02	1.2 ± 0.4	3.4 ± 0.2	5.2 ± 0.51
**TRIG (MG/DL)**							
**AMN**	7.4 ± 0.1	3 ± 1	5.25 ± 2.6	5.7 ± 2.1	2.6 ± 0.5	17.6 ± 3.4	13.7 ± 2.31
**ALL**		1.5 ± 0.5	2.2 ± 0.3	2 ± 0	3.5 ± 1.5	6.6 ± 0.5	17.5 ± 3.28
**UREA (MG/DL)**							
**AMN**	62.7 ± 3	53.9 ± 0.4	64.7 ± 15.4	60.1 ± 1.8	45.8 ± 0.9	64.9 ± 0.25	40.7 ± 2.03
**ALL**		41.8 ± 3.3	62.1 ± 13.8	64.1 ± 6.1	44.5 ± 1.9	63.7 ± 4	74.6 ± 7.43
**SG**							
**AMN**	1008 ± 0	1007.1 ± 0.210	1006.75 ± 1.3	1006.1 ± 1.9	1006.5 ± 0.5	1010.4 ± 2.6	
**ALL**		09.5 ± 0.5	1006.7 ± 1.1	1006.9 ± 1.54	1006.6 ± 0.5	1020.5 ± 1.2	

## Data Availability

Data available on request. The data presented in this study are available on request from the corresponding author. The data are not publicly available due to the originality of this work.
